# Factors that impact the timely treatment of obstetric fistulas in Malawi: The perspective of health care providers

**DOI:** 10.4102/phcfm.v11i1.1900

**Published:** 2019-04-30

**Authors:** Mwawi S. Gondwe, Pranitha Maharaj

**Affiliations:** 1 School of Built Environment and Development Studies, University of KwaZulu-Natal, Durban, South Africa

**Keywords:** obstetric fistulas, maternal health, obstetric care, delayed treatment, obstructed labour, social isolation

## Abstract

**Background:**

In sub-Saharan African countries, women face a high risk of obstetric fistulas. In Malawi, the prevalence rate is 1 per 1000 women. Studies suggest that several obstacles exist that prevent obstetric fistula patients from getting timely treatment for their condition.

**Aim:**

The aim of this article was to find out the factors that delay the timely treatment of obstetric fistula patients at Malawian hospitals.

**Setting:**

The study was conducted at the Queen Elizabeth Central Hospital, a referral hospital, situated in Blantyre, Malawi, and the findings have been generalised to all the hospitals in Malawi.

**Methods:**

An exploratory case study, employing key interview questions, was used to provide insights into why there are delays in providing treatment and care for fistula patients. Purposive sampling technique was used to identify study respondents. Key informant interviews were conducted with 16 health care personnel at a hospital in Malawi.

**Results:**

The presence of numerous cases of complicated obstetric fistula cases overwhelms the health care system in Malawi. In addition, the severe shortage of staff, lack of obstetric fistula surgery training, low staff morale, inadequate infrastructure or equipment and water scarcity in the city of Blantyre contribute towards delayed treatment of fistulas at the hospitals.

**Conclusion:**

The presence of numerous cases of obstetric fistulas is overwhelming health services, and hence there is a need for devising and implementing health policies that will motivate Malawian health personnel to undertake obstetric fistula surgery and care.

## Introduction

In 2012, there were approximately 2 million women living with obstetric fistulas globally and most of them in Asia and sub-Saharan Africa.^1^ About 50 000–100 000 new cases are reported every year globally.^2^ The data on the prevalence and incidence of obstetric fistulas for sub-Saharan African vary. Research conducted in Malawi showed that the prevalence of obstetric fistulas was 1 per 1000 women, while data for African countries show that the incidence rate was 5–10 per 1000 deliveries in remote areas of Africa.^3^

Obstetric fistula is defined as an abnormal opening in the vaginal cavity and internal organs (such as the bladder and the rectum),^4^ which results in a woman experiencing obstructed or delayed labour. Obstetric fistulas usually occur in the absence of sufficient obstetric care and when there are no provisions for giving birth through caesarean section.^5^ Delayed labour quite often leads to the death of the woman and the baby, and in the event that the woman survives, she may experience uncontrollable leaking of urine and/or stools.^6^ In many cases, injuries as a result of obstructed labour culminate in permanent disability; in some cases, it can result in long-term consequences, with some women living with the problem for over 40 years.^5^ If treatment is unavailable or not provided timely, its consequences for women are likely to be devastating. Fistulas are likely to result in the leaking of urine and/or stools.^6^ The persistent leakage can also result in damage to the vulva and thighs.^7^ Women may also experience social exclusion and isolation. They become subjects of shame, embarrassment and social humiliation and are treated as outcasts in society because of urinary or stool incontinence leading to wetness, which results in pungent odours. Studies suggest that women with fistulas are more likely to experience divorce, loss of fertility, depression and loss of earnings.^7^ This study was aimed at investigating the factors that delay women from receiving and getting treated for obstetric fistulas at health facilities in Malawi. These factors are not only unique to primigravida women but also apply to multigravida women.

Obstetric fistulas are a major public health concern in Malawi and in other sub-Saharan African countries. In Malawi, it is estimated that about 2000 new cases of obstetric fistulas are documented every year and that two to eight women in every 1000 women are at risk of developing the condition at the time of delivery.^8^ Studies suggest that age at birth influences the occurrence of fistulas.^5^ Young women who get pregnant at an early age of between 13 and 20 years are more likely to develop fistulas, and this problem becomes much more complicated if they are underweight and malnourished. A 2014 review showed that 28% of women who had developed obstetric fistulas were aged 20 years and younger.^9^ The study concluded that if the risk of early childhood birth was eliminated, there could be a reduction in the percentage of women suffering from fistulas.^9^ A study conducted in Malawi involving 3282 women, whose minimum age was 12 years, revealed that the women with obstetric fistulas were much older than what has been reported in the literature previously.^10^ In addition, they found that multiparous women were more likely to experience obstetric fistulas compared to primiparous women.^10^ They argued that this is most likely a result of poor quality of maternal health services or lack of access to emergency obstetric services because older women are less likely to experience obstructed labour than younger women.^10^ The study further posited that cultural practices may also increase the risk of developing obstetric fistulas.^10^

Women are forced to labour for days before being allowed to visit a health facility for appropriate care.^10^ In Yao culture, women are supposed to have their first birth at home and if there is prolonged labour, it is an indication that the women have not been faithful.^11^ In Burkina Faso, fistulas are seen as an indication of bad behaviour such as infidelity and disrespect for elders.^3^ In most developing countries like Malawi, obstetric morbidity such as pregnancy-related conditions, the act of delivering a baby and the period thereafter are of great concern.^12^ In these poor under-developed countries, millions of women develop fistulas because of limited availability of emergency obstetric care, which despite being inadequate is not fully utilised, accessible and often of low quality.^13^ The aim of this exploratory study was to investigate the factors for delayed treatment of obstetric fistulas of women in Malawi from the perspectives of health care providers.

## Methods

### Study design

This inquiry was retrospective and qualitative in nature as the questions required the researcher to capture perspectives relating to obstetric fistulas at Malawian hospitals.

### Setting

The study was conducted at the Queen Elizabeth Central Hospital, a referral hospital in Blantyre, Southern part of Malawi.

### Study population and sampling strategy

The respondents consisted of nurses and doctors from the Department of Obstetrics and Gynaecology of the Queen Elizabeth Central Hospital and have been working in this field for a minimum of 1 year. In total, 16 interviews were conducted. The respondents who were engaged to take part in the study were key people who are knowledgeable about issues pertaining to the treatment of obstetric fistulas and those who were in key positions in the Department of Obstetrics and Gynaecology where obstetric fistula patients are treated and taken care of in health facilities.

The researcher employed the snowball technique, a kind of purposive sampling, which entails that after a member of the targeted population is identified, he or she will refer the researcher to the others who are also knowledgeable about the subject matter being researched. The researcher held a meeting with the Head of Department of Obstetrics and Gynaecology at the Queen Elizabeth Central Hospital, who helped in identifying potential respondents. Each respondent was able to refer the researcher to another potential respondent who was also interviewed.

The researcher relied on referrals made by initial contacts who were able to recommend their peers who were eligible to participate in the study. Through this sampling technique, other stakeholders who have an interest in maternal health issues but do not work for the hospital were also consulted. These included three community workers and two faith leaders. Such a triangulation method was helpful in validating the results.

### Data collection

An exploratory case study, employing key interview questions (Appendix 1), was used to provide insights into why there are delays in providing treatment and care for obstetric fistula patients. Key interview guidelines proved vital for the study because of their ability to enable the researcher to carefully use the limited time provided by the participants, to arrange the questions in their logical order and to, if necessary, re-organise the questions. Furthermore, through key interview guidelines, the researcher was also able to observe the non-verbal communications of the participants that are not usually captured by other study instruments.

### Data analysis

The interviews were tape-recorded and transcribed using the denaturised approach whereby the content of the discussion was continuously reviewed to check for inconsistencies. Then, the data were organised depending on the questions asked. Open coding of the data was performed, which resulted in the emergence of various themes. Thematic analysis was also conducted using the grounded theory approach, which identified particular themes that emerged from the interviews. This process entailed going through the data several times, searching for themes and making comparisons with different elements, sentences and paragraphs and merging the closely related barriers into one. The data captured through non-verbal communication were analysed through conversation analysis, whereby the tape recordings and the transcriptions that followed were thoroughly inspected. The final part of the data analysis was to deduce possible and reasonable explanations of results. Then, the results were synthesised into a final report.

### Ethical considerations

Ethical approval (number BE003/15) for the study was obtained from the University of Kwazulu-Natal Ethics Committee and from the National Health Sciences Research Committee of the Ministry of Health in Malawi. There was strict confidentiality with regard to respondents’ names and the consent forms were labelled with pseudo-names and this ensured that the data collected could not be linked to individual study respondents. The research data were held in confidence and all field notes were kept in a locked file cabinet that can only be accessed by the researcher and the study supervisor.

## Results

In total, 16 interviews were conducted with a number of health personnel. The socio-demographic characteristics of the respondents are presented in Table 1. The average age of the respondents was 44 years. The youngest was 24 years and the oldest was 59 years. The majority of the respondents were women and only five were men. Most were married and only five were single. Ten of the respondents had a nursing background, two were medical doctors, three were clinical officers and one was a laboratory technician. The respondents were drawn from the obstetrics and gynaecology departments of the hospital and from non-governmental organisations that deal with maternal health in Blantyre City. Four of the respondents had more than 10 years of experience.

**TABLE 1:** Socio-demographic characteristics of the sample.

ID	Age (year)	Sex	Marital status	Occupation type	Number of years worked
P1	59	Female	Married	Enrolled nurse	11
P2	24	Female	Single	Midwife technician	2
P3	27	Female	Single	Registered nurse	4
P4	48	Female	Married	Registered nurse	9
P5	45	Female	Married	Laboratory technician	6
P6	40	Male	Married	Senior clinical officer	8
P7	52	Male	Married	Clinical officer	10
P8	34	Female	Single	Medical doctor	7
P9	38	Female	Single	Nursing midwife	7
P10	58	Male	Married	Senior clinical officer	15
P11	47	Female	Married	Enrolled midwife	7
P12	56	Female	Married	Nursing midwife	11
P13	35	Male	Single	Medical doctor	5
P14	45	Female	Married	Nurse	7
P15	57	Male	Married	Medical doctor	8
P16	42	Female	Married	Nursing midwife	8

### Barriers relating to human resources

The issues that relate to human resource were the most frequently reported factors that impact the timely treatment of obstetric fistulas at the hospital. The most common barriers were staff training, inadequate staff and a lack of morale among health care workers.

### Training

In May 2007, United Nations Population Fund conducted a 4-week training programme for surgeons as a way of empowering local medical officers in undertaking obstetric fistula repairs. These local medical officers were drawn from the Queen Elizabeth Central Hospital and district hospitals that are able to provide simple fistula repairs. Some of these districts are deemed to have high numbers of fistula cases. Those selected for training were eight in number and included five clinicians, one medical doctor, one nurse and an anaesthetist. After 3 hours of theoretical teaching, they were then taken to a theatre to observe how obstetric fistula surgeries are performed. After the observations, the trainees were given an opportunity to do the fistula repairs under strict supervision by international experts and it emerged that only one trainee was competent to do the surgery, two others were relatively competent and the rest could not manage the repairs. Their level of knowledge of obstetric fistula surgery was still not satisfactory. One respondent reiterated:

‘Before training, fistulas were not mentioned in the districts. All cases were referred to this hospital. This was my first training and at this training, it was my first time to see a fistula. This initial training was not adequate and right now we are only allowed to treat simple cases, the complicated cases are kept for the camps.’ (P12, female, aged 56)

Since then, those who are classified as very competent and competent enough are invited to the obstetric fistula camps upon the recommendation of international experts whom they understudy on how best to do obstetric fistula surgery.

Most of the personnel in the departments were trained to undertake pre- and post-fistula surgical care. However, the general consensus from the respondents is that there has not been any other training on pre- and post-surgical care of fistulas since the first one and yet there has been new research on caring for fistula patients:

‘The training on care was adequate but more needs to be done and the training needs to be done regularly and new staff should be trained on their own and there is a need to train more staff as the number of people seeking treatment is increasing.’ (P3, female, aged 27)

Unlike the normal tradition of holding the camps in May, the 2015 obstetric fistula camp was held in July and another one was held in October. The competent local experts have to go to the district hospitals and do the preliminary assessment to establish whether indeed the patient has a fistula and how complicated the case is. In case of a simple fistula, routine treatment is performed at the district hospitals by the local medical personnel who were certified as competent by international experts during the first training. The local surgeons visit other district hospitals where they conduct the surgeries. The real complicated cases are then reserved for the camps where international experts preside over the surgeries. One respondent said:

‘In the districts, the local experts do the repair of non-complicated cases of obstetric fistulas, thereby gaining more confidence to do the repair on their own and at the same time, saving resources which could have been spent on international experts.’ (P2, female, aged 24)

### Shortage of staff

The issue of shortage of skilled staff to treat obstetric fistulas was the most commonly reported barrier at the hospital, as one respondent noted:

‘There is one skilled staff who is competent enough to undertake fistula surgery and this person is not working full time as he is furthering his studies at the College of Medicine. The same person has to go to other hospitals in the region to undertake diagnosis and repair simple fistula cases. This person however can only operate on simple cases.’ (P10, male, aged 58)

There is only one local staff at the Queen Elizabeth Central Hospital, who is able to conduct fistula surgeries at the moment which is a big problem as the number of women seeking treatment and care has increased. The staff after qualifying from their colleges are reluctant to join government hospitals, preferring to work for non-governmental organisations and the private sector. Some migrate to other countries in search of better pay resulting in a shortage of staff at most Malawian hospitals. One respondent had this to say:

‘Some staff have to be temporarily deployed to district hospitals to take up positions of those that are on leave or have resigned from their posts. Some like such arrangements because they make allowances and do not want to come back. This is a burden on the other health care workers who have to fill in for those that have been deployed elsewhere.’ (P1, female, aged 59)

Acquired immunodeficiency syndrome (AIDS) has also taken a huge toll on the staff in Malawi, as many medical personnel have died and others are sick, thereby increasing the level of absenteeism at the hospitals. Hence, the management of fistulas is impacted upon by the advent of AIDS because resources are now being diverted elsewhere. Absenteeism of staff because of AIDS-related illnesses results in few health workers helping fistula patients. In addition, as a result of medical personnel dying, health facilities have lost trained personnel. Obstetric fistula camps serve as training opportunities for the local surgeons to understudy the international experts on how to undertake surgery. In some cases, staff from private hospitals and students from the College of Medicine have to be temporarily deployed to fill in for those who are absent for one reason or another. One respondent had this to say:

‘Sometimes we get employees from the private sector to come and work here and even unqualified students from the College of Medicine fill in for the medical doctors.’ (P2, female, aged 24)

The issue of staff shortage at the hospital is very critical. The management of the hospital have to temporarily employ staff from the private sector and unqualified medical doctors fill in for the scarce medical doctors. This affects the timely treatment of fistulas as there are no qualified personnel to undertake surgery and administer treatment for other ailments. In response to the problem of staff shortages, the College of Medicine of the University of Malawi, which is affiliated to the Queen Elizabeth Central Hospital, in conjunction with the United Nations Population Fund (UNFPA) started obstetric fistula camps that have been held twice every year in May and October. Obstetric fistula patients who attend these camps are from all the districts of the country and come to the hospital to be attended by international experts because they have complicated fistula cases. Community mobilisation efforts help to sensitise these patients to come to hospitals to seek treatment. As one respondent indicated:

‘Previously people never thought of fistulas as an ailment and associated it with witchcraft, adultery and disobedience to elders but the media has indeed done a good job in sensitising the communities about the availability of treatment for fistula patients.’ (P5, female, aged 45)

Before the camps, women are sensitised to come to the hospitals for screening and those diagnosed with an obstetric fistula are encouraged to come back for treatment at a later date. The sensitisation was at first done through the radio, community newspapers and through social workers, but at the moment with financial assistance from UNFPA, health surveillance assistants were recruited to help in sensitising communities about the availability of treatment for obstetric fistulas at hospitals through community mobilisation efforts. As one respondent noted:

‘The camps take approximately two weeks and patients come from all the districts. International surgeons do the surgeries during the OF [obstetric fistula] camps and the competent local personnel observe how the complicated surgeries are being done with the hope that one day they will take over.’ (P3, female, aged 27)

### Staff morale

Staff morale was the second most cited barrier among the health workers at the hospital. One respondent said:

‘The morale is very low due to low salaries and poor working conditions. We just feel like not going to work when we wake up in the morning. The problem is made worse due to the fact that we do not have equipment to work with. Most people just want to attend conferences and workshops to get allowances.’ (P5, female, aged 45)

The staff are underpaid and overworked. Some in order to supplement their income have to go and work for non-governmental organisations and private hospitals. Others do locum where they sacrifice their leave days and off-duty days to work at the hospital because of shortage of staff. Their morale is very low. The workers are frustrated further by the delay in getting their locum for certain months and also there is uncertainty with regard to continuation of locums which is being paid by the Government of Norway. The uncertainty is as a result of the Norwegian government’s intention to discontinue supporting the Malawi government in providing the funds for locums. One respondent said:

‘We received our locums for the months of March to May very late. Since the month of July there has been uncertainty with regards to locum. The Government of Norway, which was providing the locum payment, has suspended locums as from the 1st of July. We do not know how we are going to survive as the locum payment really helps us. We use it to pay for transport to work and also to buy food for our children when they are going to school.’ (P8, female, aged 34)

Hence, the low motivation of nurses and doctors contributes to poor patient management as the workers see no reason of working hard and dedicating themselves to their work because their morale is not boosted. Furthermore, one respondent said:

‘Sometimes we just lock ourselves in the offices and do not mind what is going on in the wards. Whenever there is a knock on the door or when we hear one of the patients crying in agony, that is when we go out to monitor what is happening in the wards.’ (P1, female, aged 59)

Such a situation puts the patient’s lives at risk because there is inadequate supervision of the wards by the nurses or doctors. Women in labour and obstetric fistula patients are affected by this practice as they need to be monitored. If not properly monitored, the situation can lead to women in labour having obstructed labour. Furthermore, it can easily lead to patients avoiding a particular health care centre and instead preferring those that are far away or those that are renowned for offering good care.

The fact that the medical personnel sacrifice their leaves and off-duty days in order to be paid locum rates is a problem because when they are on normal duty, they just sit in the wards and sometimes sleep during working hours whether at night duty or during day time. This is a result of the medical personnel being exhausted because they do not have proper rest. Some health care workers have worked for 10 years without going for any additional training because in most cases there is favouritism in the way they select those who go to these workshops, as told by one respondent:

‘The selection of those who are given an opportunity to get trained is not done in a fair manner. You just hear from those who went for the training or conferences telling you that they had gone for training or a conference. How they are selected nobody knows. This is demotivating to some of us who have worked here for many years but have never gone to such trainings.’ (P15, male, aged 57)

The fact that there is a suspicion of favouritism in selecting those who go for training and those who attend conferences is in itself a major demotivating factor to the hard-working health care personnel who are not recognised and rewarded accordingly. Even if one is trained and goes for further education, there is no immediate reward for those who have obtained new qualifications, as stated by one respondent:

‘I completed a degree a year ago but till now, I have not been promoted or given any bonus. I keep reminding my boss about it but nothing has been done so far to increase my salary or promote me to the next grade.’ (P10, male, aged 58)

### Barriers related to availability of supplies and facility

Other factors that were reported include the shortage of supplies, equipment, drugs and facility infrastructure that were not available and sometimes were in short supply.

### Availability of infrastructure and supplies

During fistula camps, all the necessary instruments and equipment are available, as development partners, especially the UNFPA, greatly help to ensure that every item that is required during the camps is available. A respondent stated:

‘We submit a budget to the UNFPA for the camps and then they provide all the requirements for the surgeries to be undertaken even though we have to wait for longer periods before these budgets are approved. In the budgets we include all items that are required including the pay for the international experts.’ (P14, female, aged 45)

However, after the camps, some supplies, such as repair instruments, ureteric catheters, operating tables and good light sources, needles and sutures, are not in adequate supply. This, in addition to the non-availability of international experts, prevents the routine repair of complicated obstetric fistulas. Hence, patients have to wait as there are no drugs and equipment that can be used in routine repair of the fistulas. At times, walk-in obstetric fistula patients are treated depending on the availability of treatment supplies.

There are three theatres in the Gynaecology and Obstetrics department. Two of the theatres are made available for obstetric fistula surgery during camps and one is reserved for routine emergencies. There are 10 people operated per day. In situations when there are emergencies in the labour ward and the camps are taking place, there is occasional disruption in surgery as the theatres have to be used for the emergency and thereafter the surgery continues. As one respondent said:

‘We sometimes have to temporarily suspend the fistula surgery so that we use the theatres for other complications arising from the maternity ward. After we finish treating the complications we can then continue with fistula surgery. This disturbs the whole process.’ (P13, male, aged 35)

The UNFPA also provides special food parcels to patients and other organisations help with drinks. In addition, some companies provide soya and mealie meal for the patients. The drinks and the porridge from mealie meal are highly recommended for fistula patients. The porridge provides the patients with their nutritional requirements, while the drinks help them prevent dehydration as a result of the uncontrollable leaking of urine. In addition, the autoclave and washing machines are few, making it difficult to sterilise instruments for operations and wash beddings for fistula patients. One respondent had this to say:

‘The ward cleaners wait for longer periods to have access to the autoclave and washing machines as all departments are also using the machines at the same time we are having fistula patients in the ward. This is not good as fistula patients need clean clothes.’ (P9, female, aged 48)

The fact that the clothing and beddings of fistula patients are not cleaned on time presents an unhygienic situation as fistula patients need good hygiene because their clothing and beddings smell of urine and stools. This makes the patients to have low self-esteem. Furthermore, the space at the hospital is not enough to accommodate the growing number of fistula patients who come from all over the country, as one respondent said:

‘The ward is small and can only accommodate 74 patients, resulting in fistula patients being mixed with other patients. Gynaecology patients are discharged or re-scheduled to accommodate fistula patients. There are usually close to 100 patients during fistula camps and some are accommodated in the antenatal ward. Some patients sleep on the floor on mattresses but after the surgery, they are put on beds.’ (P4, female, aged 48)

Drugs are properly stored in the available refrigerators, which are in good condition, and blood supply is available for those patients who need blood if they have been found to be in need of blood transfusion. A diesel generator is on standby in case of power outage. However, in some cases, fuel for the generator is unavailable and this makes the work very cumbersome. One respondent said:

‘Sometimes we have to depend on well-wishers who have to provide diesel for the hospital generators in time of power outage and this is very difficult as power supply in Blantyre city is unreliable.’ (P6, male, aged 40)

The non-availability of power at the hospital can jeopardise the lives of the patients, especially if they are undergoing surgery, and therefore the hospital management should ensure that the hospital has a standby generator, which has fuel in it.

The city of Blantyre is experiencing severe water shortages and as a result, hospital personnel have to keep water in buckets which has to be used in the wards. In some cases, water trucks from the Blantyre City Council come to the hospital to provide water whenever there is shortage of water in the entire city. This is not good for the hygiene of the patients, especially for fistula patients. Another respondent observed:

‘We have to share the water so that patients bath and use it in the toilets and also for cooking. This is very difficult as fistula patients require clean clothes and good hygiene. We are sometimes stranded in such circumstances as the priority is to have water for cooking.’ (P10, male, aged 58)

Fistula patients need lots of water to keep themselves clean as a coping mechanism. They need to bath regularly and wash their clothes and belongings frequently. Hence, there is a need for management at the hospital to provide enough water, especially to fistula patients, at the hospital.

### Demographic characteristics and social economic status

Some staff at the hospital are not interested in operating on fistula patients because they regard them as being poor and coming from a rural background, and they cannot afford the treatment. As a result, some consider the long fistula surgeries as a waste of their time, as shown in the following response:

‘After our group, which was the first to be trained, seven other clinical officers and five medical doctors have been trained to repair fistulas. Most of these stopped repairing fistulas because they have no interest in fistula repair as they feel it is not a rewarding job to do the surgery as most fistula patients are poor women from rural areas.’ (P6, male, aged 40)

Most women who present with fistulas are quite young and get pregnant at a young age, are divorced and have very little education. One participant said:

‘Most of these patients are aged between 15 to 28 years; their average age is 18 years, on average have two sexual partners, with most of them having been divorced and their husbands escort few of them to the hospital. Their highest education level is standard six.’ (P6, male, aged 40)

### Health status

Most of these women, when they present for fistula treatment at the hospitals, have low weight and are short; in most cases, antenatal records show that they have visited antenatal clinics twice or thrice, have never consulted other hospitals and had stillbirths.

Because of poor conditions in their homes, women are malnourished, have low weight and are unable to access antenatal care or health centres because of the long distance from their place of residence. The fact that they are malnourished makes them to be vulnerable to fistulas as their pelvises are not fully developed. They are unable to access treatment because of the poor road networks in their areas and the unavailability of health care centres:

‘Some patients are unable to walk, feel pain around the pelvis. They depend on other people to eat, drink and even dress up.’ (P15, male, aged 57)

Those that have been treated express happiness after the surgery. The women look very different after the surgery because their dignity has been restored. One respondent said:

‘Those who come for treatment get repaired and the success rate for repair is 90 percent. After repair, their faces completely change. They move around very proudly, showing a sign that their dignity has been restored. They sing songs of praise for the surgeons and nurses for bringing back their lost dignity and freedom. During follow-up visits, they sometimes bring gifts to hospital staff. However, some are fearful of the future.’ (P16, female, aged 42)

After having been repaired, the women have once again gained their dignity and are happy about their life once again even though some are not certain about the future as they are poor and do not have friends to support them financially and, in some cases, their relatives have abandoned them. They have nowhere to go and have no money to help them settle down once again. They have fear, especially with regard to their integration in their communities, families and workplaces where they had been ostracised.

## Discussion

The findings of the study show that the numerous cases of complicated obstetric fistulas are not treated by local surgeons alone as they often lack the experience or the skills to undertake this complicated procedure. If an inexperienced surgeon handles a complex case of fistulas, the results could be damaging even if an experienced surgeon is involved at a later stage.^14^ Common to the treatment of fistulas is the fact that the first case offers the best opportunity for success.^14^ If the first repair is unsuccessful, this may result in the patient and all prospective patients not trusting the health care system, which leads to them becoming reluctant to seek treatment. By engaging international experts to treat the complicated cases only, resources are saved and the local surgeons are given more experience and opportunities to undertake the surgeries themselves. This will enhance sustainability although there is still more to be done so that the local surgeons are also able to handle complicated cases of obstetric fistulas.

There is little evidence-based research to inform an inexperienced surgeon undertaking fistula repair.^15^ Obstetric fistulas do not have a standardised classification method and this undermines the health care provider’s ability to establish evidence-based practices for administering treatment.^16^ Most experts have developed their own ways of doing the repairs and this confuses the novice fistula surgeon.^16^ As many health care workers do not have a good understanding of the obstetric fistula problem, for them to undertake surgery and care becomes a cumbersome task.^17^ This compromises the quality of treatment and care that is offered to fistula patients. Furthermore, the quality of care given to patients after the obstructed labour event has an impact on the development of injuries.^5,18^ Such being the case, there is a need to impart knowledge to health care workers so that they are able to take good care of fistula patients both before and after the surgery. Such opportunities for training health care workers in pre- and post-care of fistula patients in Malawi are at the moment very few because of budgetary constraints.

As a result of the prevalence of complicated obstetric fistula cases, the health care system has adopted a system of reserving complex cases for senior surgeons who have to come from outside the country. The less difficult cases can be performed by the local surgeons, but there is a need for the senior surgeons to supervise the ureteric and urethra cases that are more complicated; hence, treatment of such cases has to be performed periodically. Such a situation makes the patient with complicated cases wait even longer when they have already waited for a long period of time, some even for more than 5 years. In this case, the women’s right to reproductive health is denied. The three delays model states that the quality of care that is promptly provided at the hospital without further delay is very important in reducing maternal mortality and morbidity.^14^ However, such quality of care is currently lacking at Malawian hospitals and this has resulted in backlogs of fistula cases.^14^

The study also unveiled a lack of morale among the health care workers. The reason for such a situation is mainly centred on problems associated with lack of career progression, lack of continuous education, lack of resources to help in the efficient discharge of duties, heavy workload, low salaries and poor working conditions. Staff morale impacts directly on staff performance and retention.^19^ It leads to poor management of patients, which eventually culminates in poor health outcomes.^20^ Staff who work in an environment in which the health outcomes are poor, and where they are overworked, are likely to abuse or degrade patients.^21^ Studies on nurse abuse of patients have pointed towards low salaries, poor conditions of service and a shortage of equipment as contributing factors.^21^

The present numbers of health care workers are insufficient to meet the health needs of the ever-growing population of fistula patients in Malawi. This is the current state of affairs in Malawi where the health care workforce is inadequate to meet the required target level of 2.28 nurses, midwives and physicians per 1000 population as recommended by the World Health Organization.^22^ A shortage of bed space and equipment, such as ureteric catheters, repair instruments, quality of operating tables and good light source, needles and suture materials, leads to a situation where surgeries cannot be performed unless they are available during fistula camps. Most of the equipment are purchased during the camps and in most cases, almost all are used. After the fistula patients in the camps have been released back into their communities, the hospitals do not have the materials to be used for the routine treatment of fistulas, and hence in cases of fistula patients suddenly walking in the hospital to seek treatment, they are turned away and told to wait for the camps.

In as much as the use of the locum has proved to be a good strategy for improving the health care worker’s remuneration, there is ample proof that work shifts of extended hours increase fatigue levels and have an impact on both patients and worker’s safety and performance.

In some places that have water scarcity, fistula patients have problems in determining what they can do with the little water that is available to them.^22^ Such a water shortage problem at the hospital makes it difficult for the women to maintain cleanliness which they also use as a coping strategy.

## Conclusion

In Malawi, patients with obstetric fistulas require urgent attention as can be demonstrated from this study which has attempted to unveil the factors that impact the timely treatment of fistula patients from the health care provider’s perspective. Patients who present with fistulas at these hospitals are not promptly treated but have to wait a long period of time before they are provided with treatment, thereby violating their rights to reproductive health. Such factors as inadequate training of staff and staff shortages, low staff morale, poor infrastructure and provision of treatment equipment and supplies, a lack of water and bad attitude of staff towards obstetric fistula patients impact the timely treatment of the patients at Malawian hospitals greatly. The findings that have been reported in this article are aimed at shedding light on the challenges faced by obstetric fistula patients in Malawi. The recommendations provide government and obstetric fistula stakeholders with solutions that can be implemented in order to treat women who suffer from obstetric fistula timely

## Appendix 1: Key informant interview schedule.

‘My name is Mwawi Gondwe and I am a student at the University of KwaZulu-Natal. I am studying for a master’s degree in Population Studies. I am working on a dissertation for this master’s course which is entitled ‘Investigation Factors that Impact on the Timely Treatment of Obstetric Fistula in Malawi: A Case Study of the Queen Elizabeth Central Hospital’. As stated to you earlier on, the theme that will come out of our discussion will be used for this dissertation and everything that we shall discuss will remain confidential.

Your contribution to this research project will be greatly appreciated and this interview will only take about 20 min.

### Introduction

- Age
- Gender
- Marital status
- Occupation
- Tell me about yourself
- For how long have you been treating fistulas?
- What has been your experience with fistula patients so far?

#### Training in fistula repair

- Did you have a formal training to treat fistula patients?
- If yes, who did the training and for how long?
- How many of you were trained to do fistula repair and how many do the actual repairing of fistulas on patients? For those who were trained to do fistula repairing, who are actually doing the job and what could be the reasons as to why the others are not doing fistula repairs despite being trained?
- Was the training adequate or did it cover all the aspects that you encounter when dealing with fistula patients?
- Is there a training manual that has been developed to be used by people who are doing fistula repairs?
- Are these trainings competency based?

#### Resources

- Do you have the necessary equipment or tools to undertake fistula surgery? What would you say about the supply of such equipment, for example, urinary bags and antibiotics?
- Do you always have the theatre at your disposal for doing the fistula repair or do you have space in the theatre to do the fistula repairs?
- Is there congestion of fistula patients at this hospital? Explain.
- Do you receive any incentive for doing the fistula repairs which require patience and lengthy periods of time?
- Do you have well-wishers apart from government supporting your work in trying to repair fistulas?

#### Challenges

What are the other challenges you encounter in your daily duties?

What has been your experience with people who present with fistula at Malawian hospitals? Are they teenagers, from rural areas or urban areas, poverty stricken, hopeless or show hope, etc.?
